# Co-Boost: boosting and guiding neuroplasticity by combining ketamine with neurofeedback-assisted learning—towards an individualised and integrated pharmaco-psychotherapy for cocaine addiction: study protocol for a randomised, placebo-controlled, double-blind, parallel-group, single-centre trial

**DOI:** 10.1186/s13063-025-08982-9

**Published:** 2025-09-25

**Authors:** Anna S. Trippel, Ladina P. Gubser, Etna J. E. Engeli, Jan Conradi, Amelie Haugg, Niklaus Zoelch, Marcus Herdener

**Affiliations:** 1 Adult Psychiatry and Psychotherapy, Psychiatric University Clinic Zurich and University of Zurich, Zurich, Switzerland; 2 Department of Child and Adolescent Psychiatry and Psychotherapy, Psychiatric University Clinic Zurich and University of Zurich, Zurich, Switzerland; 3 Institute of Forensic Medicine, University of Zurich, Zurich, Switzerland

**Keywords:** Cocaine use disorder, Addiction, Neurofeedback training, Ketamine, Spectroscopy, Real-time functional MRI, Neuroimaging, Glutamate, Reward imagery, Nucleus accumbens, Ventral tegmental area

## Abstract

**Background:**

Cocaine is the most frequently used stimulant worldwide, with increasing consumption rates in Europe. Cocaine use is associated with great harm to individuals and society. As of today, psychotherapeutic interventions for cocaine use disorder (CUD) demonstrate only modest effect sizes, and no pharmacotherapy has been approved due to gaps in understanding the disease. However, a novel pharmacotherapeutic target, i.e. glutamatergic neurotransmission, emerged from animal models of addiction. Specifically, after chronic cocaine administration, glutamate concentrations in the nucleus accumbens (NAcc) of rodents are reduced, while there is an overflow of glutamate during cue-induced cocaine-seeking. Recently, this glutamatergic imbalance has also been observed in humans with CUD. Additionally, promising findings with regard to novel psychotherapeutic approaches came from neurofeedback training (NFT) studies where participants use cognitive strategies to regulate their activity within a specific brain region based on “real-time” feedback about its activity as assessed by real-time functional magnetic resonance imaging (rt-fMRI). For example, participants with CUD successfully learned to regulate their brain activity in reward areas of the midbrain using reward imagery and to reconstitute reward sensitivity to non-drug related reinforcers like, e.g. social interactions, athletic or professional achievements. We therefore investigate the therapeutic potential and the underlying mechanisms of two interventions, a single dose of ketamine and a reward imagery rt-fMRI NFT in 120 participants with CUD.

**Methods:**

We examine a single ketamine infusion, three sessions of reward imagery rt-fMRI NFT, and the combination of those interventions contrasted to a placebo infusion or a sham NFT in 120 participants with CUD. The study is designed in a randomized, placebo-controlled, double-blind fashion with four study arms.

**Discussion:**

We expect both interventions to have a positive effect on the proportion of cocaine use days. We predict glutamate levels in the reward system to increase with the ketamine infusion and to reduce craving, a re-enhanced sensitivity towards natural rewards resulting from the rt-fMRI NFT, and synergistic effects of the combined interventions. This neurobiologically informed approach has the potential to open new avenues for the treatment of CUD through individualised and integrated pharmaco-psychotherapy.

Trial registration.

NCT06125054 ClinicalTrials.gov. Registered on October 26, 2023.

**Supplementary Information:**

The online version contains supplementary material available at 10.1186/s13063-025-08982-9.

## Administrative information

Note: the numbers in curly brackets in this protocol refer to SPIRIT checklist item numbers. The order of the items has been modified to group similar items (see http://www.equator-network.org/reporting-guidelines/spirit-2013-statement-defining-standard-protocol-items-for-clinical-trials/)

**Table:** 

Title {1}	Co-Boost: boosting and guiding neuroplasticity by combining ketamine with neurofeedback-assisted learning—towards an individualised and integrated pharmaco-psychotherapy for cocaine addiction: study protocol for a randomised, placebo-controlled, double-blind, parallel-group, single-centre trial
Trial registration {2a and 2b}.	ClinicalTrials.gov NCT06125054
Protocol version {3}	2.4, 17.09.2024
Funding {4}	Swiss National Science Foundation
Author details {5a}	Anna S. Trippel, MSc., Adult Psychiatry and Psychotherapy, Psychiatric University Clinic Zurich and University of Zurich, Switzerland, Investigator Ladina P. Gubser, MSc., Adult Psychiatry and Psychotherapy, Psychiatric University Clinic Zurich and University of Zurich, Switzerland, Investigator Etna J. E. Engeli, PhD, Adult Psychiatry and Psychotherapy, Psychiatric University Clinic Zurich and University of Zurich, Switzerland, Investigator Jan Conradi, Dr. med., Adult Psychiatry and Psychotherapy, Psychiatric University Clinic Zurich and University of Zurich, Switzerland, Investigator Amelie Haugg, PhD, Department of Child and Adolescent Psychiatry and Psychotherapy, Psychiatric University Clinic Zurich and University of Zurich, Switzerland, Collaboration Partner Niklaus Zoelch, PhD, Institute of Forensic Medicine, University of Zurich, Switzerland, Collaboration Partner Marcus Herdener, PD Dr.med., Adult Psychiatry and Psychotherapy, Psychiatric University Clinic Zurich and University of Zurich, Switzerland, Sponsor
Name and contact information for the trial sponsor {5b}	PD Dr. med. Marcus Herdener Psychiatric University Clinic Zurich and University of Zurich Adult Psychiatry and Psychotherapy Centre for Addictive Disorders Selnaustrasse 9 8001 Zürich Switzerland E-mail: marcus.herdener@bli.uzh.ch
Role of sponsor {5c}	This project is supported by a grant from the Swiss National Science Foundation to PD Dr. med. Marcus Herdener. The funders have no role in study design, data collection and analysis, decision to publish, or preparation of manuscript.

## Introduction

### Background and rationale {6a}

Cocaine is the most frequently used stimulant worldwide and its use is associated with great harm to the individual in question and their surroundings [1, 2]. Europe experienced a continuous upward trend in consumption rates over the past decade [3, 4]. In 2018, around 72,000 people in Europe sought specialised treatment for cocaine use disorder (CUD) [1]. Until today, there is no approved pharmacotherapy for CUD and psychotherapeutic interventions only show moderate effect sizes [5, 6]. This is due to (i) a limited understanding of its neurobiological underpinnings, and (ii) the fact that current psychotherapies do not specifically address the maladaptive learning processes that are associated with CUD [7, 8].

#### Diminished reward sensibility in substance use disorders

Generally, one characteristic of substance use disorders (SUD) is a remodelled reward system which is reflected in modifications within the cortico-striatal network [9–11]. This alteration in the reward system is believed to underlie maladaptive reward-seeking behaviour [12]. While exposed to substance-related stimuli, the responsiveness of the prefrontal cortex (PFC) [9–11] and nucleus accumbens (NAcc) [9, 10] is heightened, which in turn is linked to increased craving [9, 13]. On the other hand, the sensitivity of the PFC and NAcc for naturally rewarding cues (e.g. food, sex, social interaction) is reduced [14, 15]. Therefore, this shift in sensitivity to cocaine-related as compared to natural rewards [16–18] might explain why individuals with CUD choose to take substances over other rewarding activities.

#### The role of glutamate in substance use disorders

Maladaptive reward-seeking behaviour has been linked to impairments in the glutamate homeostasis in the NAcc by both human [19] and animal [20, 21] studies, pointing to a key role of glutamate signalling in SUD. In animal models of CUD, rodents show an overflow of glutamate in the NAcc when being presented with cocaine cues or cocaine probes during a withdrawal state [19, 20] that is linked with subsequent drug-seeking and self-administration. Thus, targeting the glutamatergic system is a promising pharmacological treatment approach to dampen craving and reduce cocaine use. However, no effective compound for CUD treatment has been identified for humans yet, despite extensive efforts to test glutamate affecting interventions in clinical trials [21, 22].

#### Ketamine’s effect on glutamate signalling

Ketamine, an established anaesthetic and known N-methyl D-aspartate (NMDA) receptor antagonist, affects the glutamatergic transmission in the brain [23]. In a preliminary study with rodents, investigators found a significant increase in glutamate in the NAcc after a ketamine injection [24]. Besides its anaesthetic properties, ketamine has antidepressant qualities [25] and shows preliminary promising results in the treatment of SUDs [26–29]: in both alcohol use disorder and CUD, less craving was observed and the number of days of abstinence was greater for individuals in the ketamine group compared to the control group. To gain a better understanding of ketamine’s mechanism of action, proton magnetic resonance spectroscopy (^1^H-MRS) provides in vivo and non-invasive insight into neurometabolism [30]. With a tailored approach, it is possible to measure glutamate levels even in small subcortical regions such as the NAcc [31]. Therefore, we will investigate if ketamine changes glutamate levels in patients with CUD in the NAcc together with its impact on the cue-induced increase of glutamatergic transmission associated with craving states. In addition, we will test if baseline levels of glutamate in the NAcc might serve as a potential biomarker predicting the clinical outcomes of the study and therefore are capable of assisting a stratified treatment approach in SUD. Furthermore, in addition to its direct effects on glutamatergic neurotransmission, ketamine is thought to increase synaptic neuroplasticity by promoting long-term potentiation (LTP) and inhibiting long-term depression (LTD) in neurons [32, 33], key substrates of learning- and memory-related synaptic plasticity typically involving the activation of NMDA and alpha-amino-3-hydroxy-5-methyl-4-isoxazolepropionic acid (AMPA) receptors. Via AMPA activation and a subsequent augmented release of brain-derived neurotrophic factor (BDNF) [34–37] and mTOR [35, 38], ketamine promotes protein synthesis, synaptogenesis, and strengthening of existing synapses [37–40]—all prerequisites of neuroplasticity. This supports the notion that ketamine opens a window of opportunity for learning and plasticity.

#### Modulation of reward learning by means of neurofeedback

Neurofeedback training (NFT) based on real-time fMRI (rt-fMRI) assists people to acquire and optimise individualised cognitive strategies to control activity within a specific brain region based on “real-time” feedback about the brain’s activity as assessed by fMRI [41]. This method has been successfully applied not only in healthy participants but also in individuals with SUD [42]. Furthermore, reward imagery rt-fMRI NFT was successfully applied by individuals with CUD for self-regulating small midbrain structures within the reward circuitry like the ventral tegmental area (VTA) [43] and the NAcc [41, 44]. In a previous pilot study, we could show that regular cocaine users can learn to self-regulate activity within the midbrain region with the assistance of rt-fMRI NFT, comparable to healthy participants. Interestingly, the ability to activate VTA by means of non-drug-related rewarding imagery was negatively correlated with cocaine craving and lifetime use of cocaine [43]. Therefore, the results suggest a potential therapeutic benefit of this approach for treating CUD by helping to reestablish sensitivity to non-drug-related rewards and to potentially reduce drug-taking behaviour. A reduced self-regulation in those with more severe CUD and a lack of transfer effects after a single training session highlights the need for repeated sessions and therefore more training and an optimised NFT paradigm. With those changes in the NFT setup, we believe rt-fMRI NFT of reward-related brain regions that enhances the sensitivity of non-substance-related rewards constitutes a novel and promising therapeutic approach for CUD.

### Objectives {7}

The overall objective of this study is to test the therapeutic effects and mechanisms of action of (i) a single dose of ketamine, (ii) a reward imagery rt-fMRI NFT, and (iii) the combination of those interventions compared to placebo conditions in individuals with CUD.

Therapeutic effects of the interventions—alone and combined—are measured via the investigation of changes in cocaine use assessed by questionnaires.

Mechanistic effects of the interventions—alone and combined—are measured via the investigation of (a) ketamine-induced changes in accumbal glutamate levels with ^1^H-MRS.

and (b) effects of the reward imagery rt-fMRI NFT on reward processing.

### Trial design {8}

**Fig. 1:**
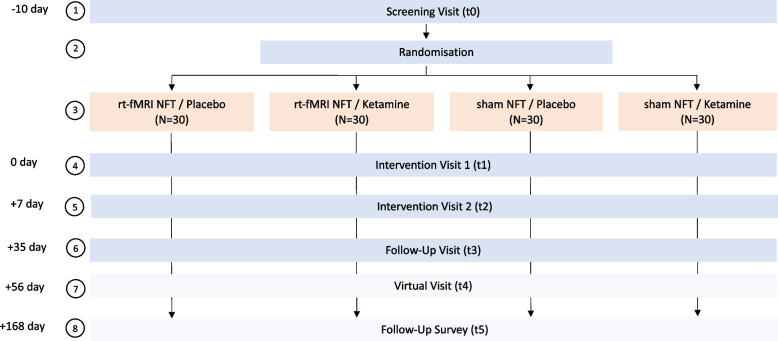
Study flow chart. Illustrating timeline and group assignments for participants in the clinical trial

The trial adopts a randomised, placebo-controlled, double-blind, parallel-group, single-centre design with a superiority framework. The treatment combination among the four arms, each consisting of 30 participants, is depicted in Fig. 1.

## Methods: participants, interventions and outcomes

Study methods have been reported in accordance with the Standard Protocol Items: Recommendations for Interventional Trials (SPIRIT) reporting guidelines [45] (see Additional file 1).

### Study setting {9}

The first four visits take place on-site at the Psychiatric University Clinic Zurich in Switzerland, followed by a fifth visit conducted remotely via video call, and the final visit involves completing an online survey.

### Eligibility criteria {10}

#### Inclusion criteria

- Informed consent as documented by signature
- Male and female cocaine users 18 to 55 years of age
- DSM-5 diagnosis of CUD
- Willingness to comply with the study protocol as explained by the investigator
- Normal level of language comprehension (German or Swiss-German)

#### Exclusion criteria

- Current or lifetime psychotic disorders
- History of severe substance-induced psychosis
- Current or lifetime bipolar I or II disorders
- Current suicidality
- Previous suicide attempts during the last 2 years
- Current severe alcohol use disorder
- Current severe cannabis use disorder
- Current moderate or severe stimulant use disorder (other than cocaine)
- Current moderate or severe benzodiazepine use disorder
- Current opioid use disorder
- First-degree relatives with psychotic disorders
- Beck Depression Inventory Score greater than 25
- Unmedicated or unstable hypertension
- Severe illness (e.g. myocardial ischemia or arrythmias, severe pulmonary secretions, glaucoma, congestive heart failure or angina, significant renal or hepatic impairment)
- Acute infection (e.g. pulmonary or upper respiratory tract infection)
- Insufficient treated or uncorrected hyperthyroidism
- Severe CNS-related traumas or disorders (e.g. stroke, cerebral trauma with loss of consciousness over more than 24 h, epilepsy)
- Increased intracranial pressure
- Medication directly affecting glutamate signalling (e.g. anticonvulsant medication)
- Any unstable psychoactive medication (no changes in compounds within the last 4 weeks before the start of study)
- Pregnancy or lactation
- Women of childbearing potential with no use of medically accepted contraceptives (e.g. condoms, contraceptive diaphragm, birth control pill, hormone injection, intrauterine device)
- BMI > 35
- Allergy, hypersensitivity, or other adverse reaction to previous use of ketamine
- Contradictions to magnetic resonance imaging
- Concurrent participation in other clinical study

### Who will take informed consent? {26a}

Participants receive the study information and informed consent at least 24 h before the first visit (t0). Delegated investigators obtain written informed consent from all eligible participants on the first study visit (t0) before conducting any trial-related procedures and before collecting any data. Furthermore, participants will receive a copy of the signed consent form.

### Additional consent provisions for collection and use of participant data and biological specimens {26b}

There is a separate consent form for the further assessment of biomarkers in blood plasma samples and optional secondary use of data.

## Interventions

### Explanation for the choice of comparators {6b}

Two forms of interventions are combined and compared: a single dose of ketamine and a reward imagery rt-fMRI NFT. Both interventions are placebo-controlled, which leads to four study arms with the following intervention combinations: (i) rt-fMRI NFT/placebo, (ii) rt-fMRI NFT/ketamine, (iii) sham NFT/placebo, and (iv) sham NFT/ketamine. This study group allocation allows for comparing each active treatment to its placebo treatment and, in addition, the combination of both interventions to a placebo study arm, that is, study arm (iii) sham NFT/placebo.

### Intervention description {11a}

#### Ketamine

On the screening visit (t0), all participants are prepared for the ketamine/placebo infusion by clarifying questions and providing information, and thereby, creating a safe framework for the subsequent intervention at intervention visit I (t1). During this preparation, participants are encouraged to develop a guiding motto that can help if challenging experiences arise during the ketamine/placebo infusion (e.g. breathe deeply, be curious, observe without judgment, surrender). Ultimately, the modifications they intend to implement with regards to their cocaine consumption over the course of the study period (i.e. specific goals for the reduction of cocaine use) are established together. For safety reasons, all participants are instructed to abstain from using illegal substances for 3 days and from alcohol for 2 days before intervention visit I (t1).

If the participant has been found eligible for study participation, the participant is allocated to one of the four study arms: (i) rt-fMRI NFT/placebo, (ii) rt-fMRI NFT/ketamine, (iii) sham NFT/placebo, and (iv) sham NFT/ketamine. The hospital pharmacy receives an automatic email generated by the data management system with information about the study arm allocation and prepares accordingly either an infusion bag of 1 mg/ml ketamine hydrochloride (Ketalar®) in 0.9% saline or a 0.9% saline solution as placebo. Both solutions have a total volume of 120 ml to ensure a standardised administration via the infusion pump (Space® Infusion Pump). The dose of ketamine is administered based on body weight at a rate of 0.71 mg/kg, given intravenously over 40 min. The dosage and route of administration of ketamine correspond to those used in previous studies investigating ketamine’s efficacy in CUD [26, 46]. Doses are generally higher in studies with SUD than in studies with depression, based on findings that individuals with alcohol use disorder may show cross-tolerance to ketamine [47]. The dose of 0.71 mg/kg or higher has consistently shown to have a good tolerability in populations with SUD [26, 46, 48–52].

**Fig. 2:**
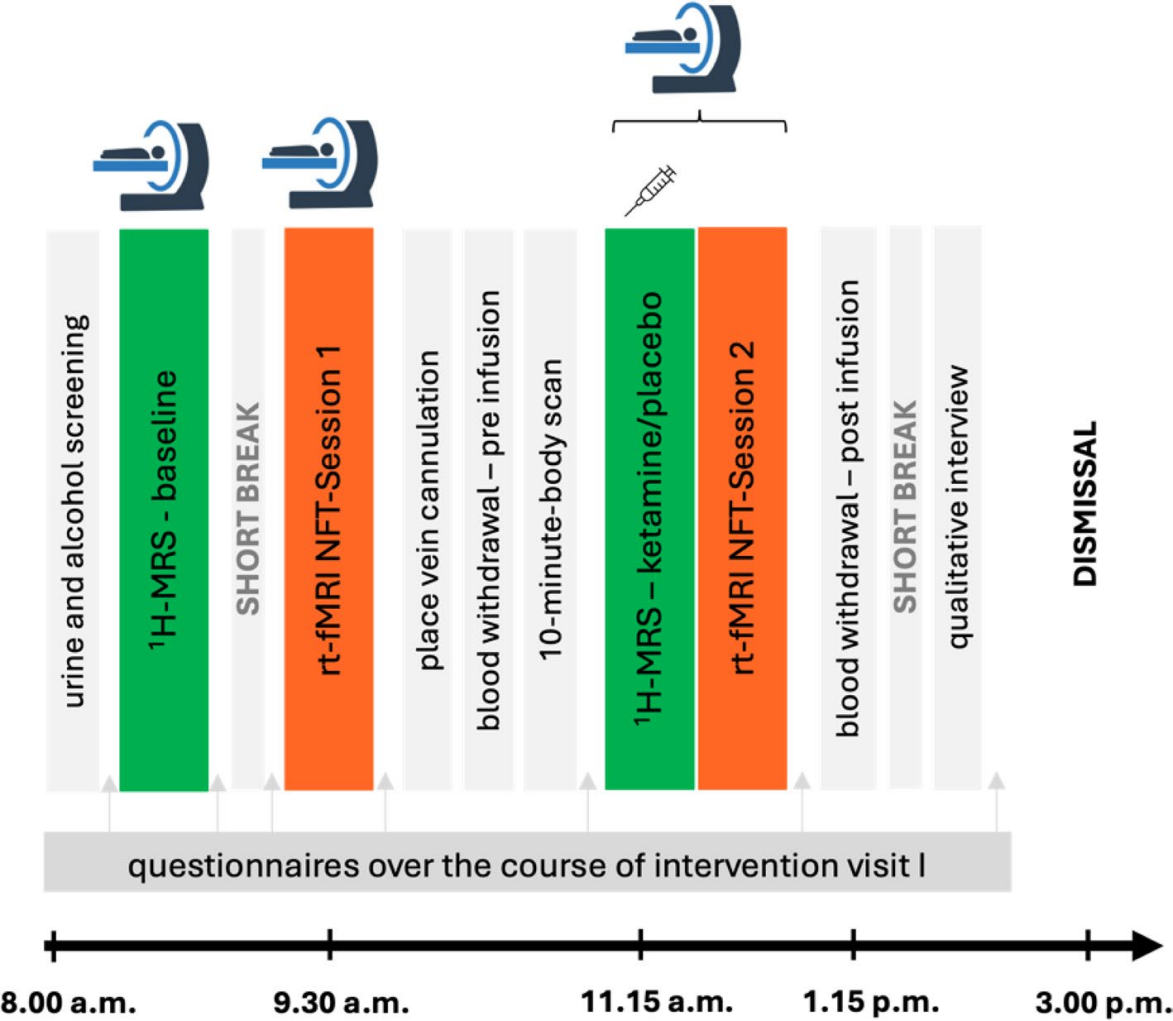
Timeline for intervention visit I (t1). Participants perform two ^1^H-MRS measurements (baseline and during the ketamine/placebo infusion), and two reward imagery rt-fMRI NFT sessions (before and after the ketamine/placebo infusion). Blood withdrawal takes place pre and post infusion. Time points for questionnaires are indicated with grey arrows

On intervention visit I (t1), when the ketamine/placebo infusion is administered, at first, baseline magnetic resonance (MR) measures are performed as a reference for the ketamine-induced changes, followed by identical MR measures that are performed during the ketamine/placebo infusion to capture glutamate levels over the course of the 40-min infusion. The MR measures include a ^1^H-MRS sequence (40 min) and during all MR measurements, participants are listening to an instrumental music playlist. Following the baseline MR measurements, participants take a short break outside of the MR scanner before they perform the first session of reward imagery rt-fMRI NFT (see intervention description—reward imagery rt-fMRI NFT). After the rt-fMRI NFT, participants have another short break outside of the MR scanner and have a peripheral vein cannula placed on their forearm. Before the start of the ketamine/placebo infusion, participants perform a 10-min audio-guided mindfulness-based exercise (body scan) to shift their focus inwards. Then, the infusion is started simultaneously with the ^1^H-MRS measurement. After 10 min of steady-rate infusion, a bolus at half of the total dose is administered for the next 10 min. The infusion then returns to a steady-rate for the remaining 20 min. After the infusion, participants perform the second reward imagery rt-fMRI NFT right afterwards. The whole procedure for intervention visit I (t1) is depicted in Fig. 2.

Throughout the whole day, participants are asked about their cocaine craving and affective state on visual analogue scales. In addition, following the infusion, they answer questions about subjective effects during the administration, and a qualitative interview is conducted at the end of intervention visit I (t1) to capture the personal experiences during the ketamine/placebo infusion. Medical coverage is provided until 3 h post-infusion, and participants are discharged only after they have been screened for suicidality. Besides blood pressure monitoring pre- and post-infusion, the following safety measures are taken on intervention visit I (t1): a drug urine quick test, an alcohol breath test, and individuals of childbearing potential must take a urine pregnancy quick test.

For further serum analysis of the neuroplasticity marker BDNF, blood is withdrawn 30 min prior and 2 h post infusion.

During the intervention visit II (t2), which will be held 7 to 14 days after the intervention visit I (t1) an ^1^H-MRS measurement to assess accumbal glutamate levels will be conducted during a craving paradigm, namely a video of 22.5 min that reliably induces craving in cocaine users [19].

#### Reward imagery rt-fMRI NFT

As with ketamine, as soon as the participant is randomised, an automatic email is sent by the data management system to the study team with the blinded rt-fMRI NFT code (i.e. group “dog” or group “cat”). In total, each participant takes part in three reward imagery rt-fMRI NFT sessions within the study. The first two sessions take place at intervention visit I (t1). One before and one after the ketamine/placebo infusion, and the third training session takes place at intervention visit II (t2).

As preparatory training, participants are asked to do the Prospective Imagery Task [53] during the screening visit (t0), where they need to imagine rewarding situations.

**Fig. 3:**
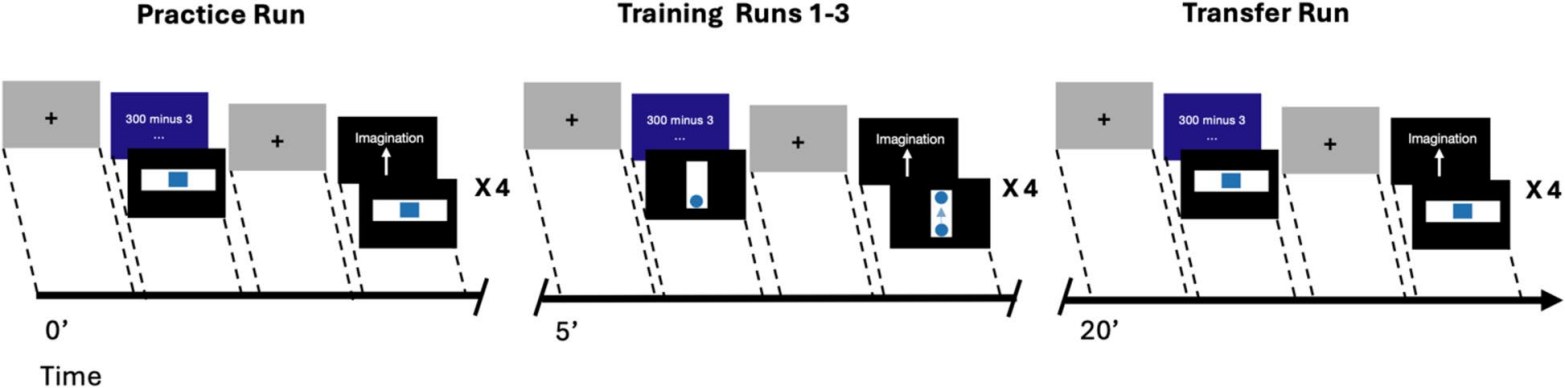
Task design (adapted from [41] and [84]). During training session one and three, each participant undergoes five runs, each one composed of “Count” (20 s) followed by “Imagination” (20 s), then repeated four times. The first and last runs (practice and transfer run) only showed instructions with no visual feedback. During the three training runs, we instruct participants to use rewarding non-drug imagery to raise the ball during “Imagination” and counting backwards to keep the ball down during “Count”

As a first step at the intervention visit I (t1), participants receive a standardised oral instruction about the following reward imagery rt-fMRI NFT task. The instruction is identical for the sham and for the real rt-fMRI NFT. The experimenter explains that one is capable of increasing neural activity in brain regions important for reward processing by using positive imagery. This positive imagination can contain any rewarding situation (e.g. sexual experience, anticipation of food, …). The only two rules are that the reward imagery must not be substance-related, and participants are not allowed to use strategies that involve music, such as playing an instrument or listening to music, due to possible activation of the Heschl’s gyrus, which is the chosen sham brain region. The reward imagery rt-fMRI NFT paradigm takes around 25 min. The paradigm for training one and three comprises five runs: a practice run without feedback, followed by three training runs with feedback, and at last a transfer run without feedback. Training session two comprises only three practice runs with feedback. Within each run, there are four blocks where participants are alternately instructed to either upregulate their activity in the target region by using a strategy of positive imagination or by counting backward to keep the activity in the target region at rest. The activity of the target region is visualised to the participant in the form of a bar with a ball that moves upwards when positively activated or stays at the bottom when unchanged, respectively, to provide feedback about the current performance in regulating one’s own activity. The feedback presentation is in an intermittent fashion right after each block (i.e. “positive imagination” and “counting backward”) (illustrated in Fig. 3). We have chosen intermittent feedback, due to reduced cognitive demands and confounding effects on reward-related activity [54].

At the beginning of each training session, either the VTA mask (probabilistic mask as defined by [55]) or the mask of the Heschl’s gyrus, the sham region (AAL Atlas) is co-registered with the functional MR images to minimise displacement artifacts due to head movements. Despite the displayed feedback, which is provided from different brain regions, all procedures and instructions are identical for the participants who receive sham NFT as for those who receive the real rt-fMRI NFT. The intervention is double-blinded.

The feedback signal is calculated with OpenNFT [56], a software package for rt-fMRI NFT. The feedback signal is estimated based on activity levels in the respective target region (i.e. VTA or Heschl’s gyrus) as the area under the curve (AUC) between rest and regulation. The feedback is calculated as follows: double of the forehand-related rest block compared to the regulation block (i.e. “positive imagination” and “counting backward”).

Between each run, participants answer a short in-between questionnaire that includes subjective questions about their feelings and motivation during the last run, the applied strategies, the contingency between received feedback and used strategy, and whether they were looking at the cross during the rest block (see Additional file 2, Table 1).

For the control condition, sham NFT, an alternative brain region was chosen. The signal for the feedback is extracted from the Heschl’s gyrus mask (AAL Atlas).

When the reward imagery rt-fMRI NFT paradigm is complete, participants answer a debriefing questionnaire outside of the MR scanner that includes subjective questions about how they liked it, how good their skills were in general, the strategies used, whether they used substance-related or music-related strategies, and whether they felt an improvement (see Additional file 2, Table 2).

The second training session takes place right after the finished ketamine/placebo infusion during the intervention visit I (t1). During this training session, the participant does not need to answer the questions in between. The procedure of the third training session during intervention visit II (t2) is the same as for the training session one.

### Criteria for discontinuing or modifying allocated interventions {11b}

Each participant is informed that the participation in the study is voluntary and that he/she may withdraw from the study at any time. The study may also be discontinued by the investigator if the participant does not comply with the study protocol, if further study participation would bear a risk to the health of the participant, or in case of pregnancy.

No modifications to the allocated treatment will be made.

### Strategies to improve adherence to interventions {11c}

Both interventions, ketamine and reward imagery rt-fMRI NFT, are carried out on site. Therefore, the investigator monitors the adherence to both interventions directly. The ketamine/placebo infusion is administered to the participant by medically trained staff at the intervention visit I (t1). Reward imagery rt-fMRI NFT is closely guided by the investigators. The compliance to the application for ecological momentary assessment (EMA) is continuously monitored. In case of an insufficient response rate in the EMA, the participant will be contacted by an investigator. Additionally, a motivational monetary bonus is given if the participant answered at least 80% of the EMA requests. Adherence to the study protocol is continuously monitored by recording concomitant medication, urine pregnancy testing, and drug screening with urine analysis and questionnaires.

### Relevant concomitant care permitted or prohibited during the trial {11d}

#### Concomitant therapy

Psychotherapeutic or psychological treatment from third parties needs to be suspended between intervention visit I (t1) and intervention visit II (t2), which is a minimum of 7 and a maximum of 14 days. Moreover, participants are instructed to inform the study personnel about therapies or operations that take place during the first four visits of the study.

#### Prohibited medications

Medications directly affecting glutamate signalling, e.g. anticonvulsant drugs as well as ketamine use during the study are not desirable and are monitored throughout the study. In addition, all new regular uses or dose changes of already existing concomitant medication are also monitored throughout the study.

### Provisions for post-trial care {30}

All participants are advised to contact the study team at any point during the study period if there are questions or concerns about the intervention or the study. Given the absence of anticipated harm due to study participation, no specific provision for post-trial or ancillary care is foreseen by the study protocol. However, any damage developed in relation to study participation is covered by the insurance.

### Outcomes {12}

All outcomes are summarised in Table 3 (see Additional file 2, Table 3) and their timepoints are outlined in Table 1.

**Table 1:** Participant timeline

Study period						
	Screening visit	Intervention visit I	Intervention visit II	Follow-up visit	Virtual visit	Follow-up survey
Visit	t0	t1	t2	t3	t4	t5
Time	− 84 days to − 10 days	0	+ 7 days to + 14 days	+ 35 days to + 49 days	+ 56 days (± 7 days)	+ 168 days (± 14 days)
Patient information and informed consent	x					
In-/exclusion criteria	x					
Demographics	x					
Body weight	x					
Electrocardiogram	x					
Mini-International Neuropsychiatric Interview, DSM-IV	x					
Routine laboratory tests	x					
Randomisation	x					
Pulse and blood pressure	x	x				
Urine pregnancy test (women only)		x	x			
Laboratory test for neuroplasticity marker	x	x				
Drug urine quick test		x				
Drug urine laboratory analysis	x	x	x	x		
Concomitant medication form	x	x	x	x		
Interview for psychotropic drug consumption	x	x	x	x	x	
Timeline follow back, version for cocaine use	x	x	x	x	x	x
Visual analogue scale for change	x	x	x	x	x	x
Obsessive compulsive cocaine use scale	x		x	x	x	x
Beck Depression Inventory	x		x	x		
Domains Of Pleasure Scale	x		x	x	x	x
Trait Hedonic Capacity Scale	x		x	x	x	x
Rosenberg Self-esteem Scale	x		x	x		x
Questionnaire for Self-Efficacy, Optimism, and Pessimism	x		x	x		x
Stress Questionnaire, Subscale of Stress & Coping Inventory	x			x		x
Generalised expectancies for Negative Mood Regulation	x			x		
Prospective Imagery Task	x					
Study Medication Administration		x				
Hood’s Mysticism Scale		x				
Five-Dimension Altered States of Consciousness Questionnaire		x				
Neurofeedback in between Questionnaire		x	x			
Neurofeedback Post Questionnaire		x	x			
MRI Safety Form		x	x			
Magnetic Resonance Spectroscopy		x	x			
Real Time Functional Magnetic Resonance Neurofeedback Training		x	x			
Visual analogue scale for affective state		x	x			
Visual analogue scale for cocaine craving		x	x			
Qualitative interview		x	x	x		
Serious adverse events		x	x	x	x	

#### Primary outcomes

This study primarily seeks to determine whether the interventions, namely the single ketamine infusion, the reward imagery rt-fMRI NFT, and those two interventions combined compared to the placebo conditions show a therapeutic effect by reducing the proportion of cocaine use days. The primary efficacy value is the proportion of cocaine use days between intervention groups on follow-up visit (t3). This is measured using the timeline follow back for cocaine use, which is a standardised and validated instrument to assess cocaine use [57].

In addition to the treatment outcome, the mechanism of action of the interventions is assessed. Firstly, changes in accumbal glutamate levels in the NAcc on the intervention visit I (t1) before and during the ketamine/placebo infusion and on the following intervention visit II (t2), approximately one week after the ketamine/placebo administration are measured by using ^1^H-MRS. Secondly, the mechanistic effects of the reward imagery rt-fMRI NFT are assessed by measuring its clinically relevant transfer effect, which is defined as the difference in performance on the intervention visit I (t1) and the intervention visit II (t2) measured in the percent signal change in the NFT region by using rt-fMRI.

#### Secondary outcomes

Furthermore, psychological and neurobiological changes that are linked with CUD and might result from both interventions are of interest:

- Changes in cocaine use measured by laboratory urine analysis of cocaine and cocaine metabolites [58, 59]. on t0 and t3
- Changes in cocaine craving measured by the Obsessive Compulsive Cocaine Use Scale [60]. (OCCUS) on t0, t2, t3, t4, and t5
- Changes in severity of CUD by using the OCCUS [60]. on t0, t2, t3, t4, and t5
- Changes in motivation for change regarding cocaine use behaviour by using a visual analogue scale (VAS) on t0, t1, t2, t3, t4, and t5
- Changes in reward sensitivity by using the Trait Hedonic Capacity Scale [61] (THC) and Domains of Pleasure Scale (Masselink M, Roekel EV, Heininga VE, Vrijen VE, Oldehinkel AJ: Domains Of Pleasure Scale (DOPS): assessing level and change in pleasure across domains, submitted). (DOPS) on t0, t2, t3, t4, and t5
- Changes in emotion regulation skills by using the Negative Mood Regulation Expectancies scale [62]. (NMR) on t0 and t4
- Changes in depressive symptoms by using the Beck Depression Inventory [63]. (BDI) on t0, t2, and t3
- Changes in stress coping measured by the subscale of the Stress & Coping inventory [64]. on t0, t3, and t5
- Changes in self-esteem measured by the Rosenberg Self-Esteem Scale [65]. (RSES) on t0, t2, t3, and t5
- Changes in self-efficacy measured by the questionnaire for self-efficacy, optimism, and pessimism [66]. on t0, t2, t3, and t5
- Subjective effects of the ketamine/placebo infusion measured by the 5-Dimension Altered State of Consciousness Questionnaire [67] (Reissmann S, Hartmann M, Kist A, Liechti ME, Stocker K: Assessing ketamine’s acute psychoactive properties with an enhanced scale for altered states of consciousness: a repeated-infusions study in a depressed patient, submitted) (5D-ASC) and Hood’s Mysticism Scale [68]. (HMS) after the infusion on t1
- Vividness of mental imagery measured by the Prospective Imagery Task [69]. (PIT) on t0
- Individual imaging strategies measured by the Neurofeedback Post Questionnaire [70]. as well as the Neurofeedback in between Questionnaires on t1 and t2
- Current cocaine craving measured by a VAS during t1 and t2
- Current affect measured by a VAS during t1 and t2
- Glutamate changes during a craving-inducing video measured by.^1^H-MRS on t3
- Changes in plasma BDNF measured by an immunoassay kit pre to post ketamine/placebo infusion on t1
- Course of real time cocaine use, cocaine craving, affect, and reward sensitivity by using ecological momentary assessment (EMA) [71]. between the first five visits (t0, t1, t2, t3, and t4)

#### Other outcomes of interest

An electrocardiogram (ECG) and a clinical interview will be conducted and evaluated at the screening visit (t0) to ensure mental and physical health. To capture the consumption pattern of cocaine and potentially other psychoactive substances, the Interview for Psychotropic Drug Consumption [72] will be conducted on screening visit (t0), intervention visit I (t1), intervention visit II (t2), follow-up visit (t3), and virtual visit (t4).

On intervention visits I (t1) and II (t2) and the follow-up visit (t3), we are conducting qualitative interviews to assess acute and sustained effects of the interventions on the subjective experience and the predictive value of those effects [73, 74].

#### Safety outcomes

The following outcomes are measured on the screening visit (t0) for safety and eligibility reasons:

- Vital signs measured by a blood pressure monitor
- Current suicidality by using the suicidality questions of the MINI [75].
- Blood parameters by using routine laboratory analysis

On intervention visit I (t1) vital signs are measured pre and post infusion at four different time points. Individuals of childbearing potential must take a pregnancy test on intervention visit I (t1) and II (t2) before the MR measurement, and all participants must perform a drug urine quick test on both intervention visits before performing any interventions.

On every on-site visit, participants are screened for current suicidality by using the questions from the MINI [75].

### Participant timeline {13}

The schedule for data collection and visits is shown in Table 1. After initial establishment of contact, interested candidates are invited for a screening visit (t0), where the eligibility criteria are checked, and suitable participants are randomly assigned to one of the four study arms. The assigned treatment intervention is performed at intervention visit I (t1) and II (t2). The follow-up visit (t3) assesses first effects of the intervention on behavioural measures. Between each of the first five visits, participants are asked to use an EMA application [71] on their smartphones. To measure long-term effects, there is a virtual visit (t4) and a follow-up survey (t5) scheduled.

### Sample size {14}

Sample size was determined by power analysis (G*Power 3.1) and primarily focused on ^1^H-MRS, the most critical procedure in this project to estimate the required number of participants to detect between- and within-subject effects as well as interaction of those. Prior ^1^H-MRS studies that reported significant glutamate differences with effect sizes measured in psychiatric populations within the striatum showed effect sizes of *d* = 1.6 [76], *d* = 0.89 [12], *d* = 1.93 [77], or measured in other brain regions in tobacco use disorder *d* = 1.03 [78]. Thus, assuming a conservative effect size of *d* = 0.80, an α-error probability of 5%, and a conservative power estimation of 80%, a total sample size of 21 individuals with CUD per group (total sample of 84) would be required to detect a significant group effect between experimental and control groups. Again, assuming an effect size of *d* = 0.80 (*f* = 0.40, respectively) for within-subject comparisons before and after intervention, an α-error probability of 5%, and a conservative power estimation of 80%, indicates that a total number of 111 subjects would be necessary to detect a significant within-factor effect in an ANCOVA model. To account for potential dropout, we will recruit in total 120 participants.

### Recruitment {15}

Recruiting takes place throughout the study timeline. Participants are recruited through different channels, for instance, from the Centre for Addictive Disorders, Psychiatric University Hospital Zurich, University of Zurich, and other clinical institutions specialised in the treatment of SUD. Furthermore, we advertise the study via flyers, advertisements, and on social media (e.g. Instagram, Facebook). Interested persons can also register on the study website, which was specially set up for the study, and are then contacted by telephone by an investigator. During the first telephone contact, potential candidates are pre-screened regarding inclusion and exclusion criteria. Those who pass the telephone screening are invited to the screening visit (t0).

## Assignment of interventions: allocation

### Sequence generation {16a}

Each participant gets a subject code, which is generated by the investigator at study inclusion and is individually allocated. The randomisation of the four study arms is counter-balanced with a 1:1:1:1 allocation ratio as per a computer-generated randomisation schedule using the method of minimisation, with age, gender, and severity of CUD based on the assessment of the DSM-5 criteria by the structural interview MINI [75] at the screening visit (t0) (mild and moderate vs. severe) as binary minimisation variables.

### Concealment mechanism {16b}

Each participant is randomly assigned to one of the four treatment arms. To ensure comparable sample size and sample characteristics in the four treatment arms, a stratified randomisation is performed by first grouping each participant into a stratum based on age, gender, and severity of CUD. The treatment allocation is performed automatically by the data management system, where the allocation list is stored by a person who is not involved in the study procedures and has no interaction with participants. Before study completion, all members of the study team who conduct study procedures with participants are blinded and have no access to the allocation list in the data management system.

### Implementation {16c}

As soon as a participant is found eligible for study participation, the data management system sends an automatic email to the Medical Centre Pharmacy of the Psychiatric Hospital Zurich informing them about the pharmacological treatment, i.e. ketamine or placebo. Since the Medical Centre Pharmacy prepares the infusion according to the treatment allocation in the received email, they are the only members of the study team who know of the pharmacological intervention. However, the staff of the Medical Centre Pharmacy never interacts with participants. The infusion bag will be of identical appearance, including colour, shape, size, and texture.

A second email is sent by the data management system to the investigators of the study team with an allocation code for the reward imagery rt-fMRI NFT. Specifically, the code names “cat” and “dog” correspond each to a different anatomical mask that is used either for the real rt-fMRI NFT or the sham NFT, which will be used for the NFT software program OpenNFT [56] to specify the correct region for the feedback calculation. This allows the investigators to implement the reward imagery rt-fMRI NFT according to the correct NFT allocation without knowing whether it is the real rt-fMRI NFT or the sham NFT. The codes for the reward imagery rt-fMRI NFT allocation have been assigned by a person who is not involved in the study procedures and has no interaction with participants.

## Assignment of interventions: blinding

### Who will be blinded {17a}

The participants, investigators, and the sponsor-investigator are masked to treatment allocation during the entire study duration. To guarantee masking throughout the study, the treatment allocation is kept in a confidential key-locked place on-site and is only accessible by a staff member of the Psychiatric University Hospital Zurich not involved in the study.

### Procedure for unblinding if needed {17b}

In circumstances under which unblinding is permissible, e.g. in case of a medical emergency, an Emergency Code Break in sealed envelopes is available to the investigators. This Code Break should be opened only in emergencies when the identity of the investigational product must be known by the investigator to provide appropriate medical treatment.

## Data collection and management

### Plans for assessment and collection of outcomes {18a}

The study team monitors data in real time to ensure complete data collection for all participants, including those who discontinue treatment. Relevant data from the participant, such as eligibility criteria, demographics, height, weight, vitals, MINI [79], concomitant medications, interview for psychotropic drug consumption, and different questionnaires (see specific information in Table 1) are entered directly by the participant or investigator into the data management system at each visit. Additionally, any data that cannot directly be entered into the system is manually added by trained study personnel and verified through a double-check process. The investigators must maintain source documents for each participant in the study, consisting of demographic information, medical and psychological screening, for example, ECG assessments, laboratory tests, and the original signed informed consent form. All original paper–pencil based data and source data documents are stored in a physical participant folder, and a copy is stored electronically on the password-protected study servers provided by the Psychiatric University Hospital Zurich. Also, the collected ^1^H-MRS and reward imagery rt-fMRI NFT data for each visit are stored on the study server. Data which is collected with the EMA application is stored in an instance of Google’s cloud-based Firestore database in an anonymised fashion [71]. In addition, a backup copy of the EMA data is saved on the study server.

For collecting the data, only standardised questionnaires and tested procedures are used.

### Plans to promote participant retention and complete follow-up {18b}

A participant is considered lost to follow-up if one fails to return for one study visit and is no longer available for upcoming visits or the study staff is unable to contact the participant after at least five attempts. Furthermore, the study team is required to document all attempts to obtain follow-up data.

### Data management {19}

Data management is carried out according to Good Clinical Practice (GCP). A secure data management system (REDCap) is used to collect and manage the collected data, such as questionnaires. Furthermore, a password-protected study server provided by the Psychiatric University Hospital Zurich is used as storage for ^1^H-MRS and reward imagery rt-fMRI NFT data. A detailed description of the data management procedure is found in chapter 18a (plans for assessment and collection of outcomes).

### Confidentiality {27}

All personally identifying information and health information, which is needed for recruitment, participation, and tracking, is securely stored by project personnel on a secured server with limited access. Only trained and delegated study personnel have access to those files and systems. Data entry is only performed by persons authorised to have access. All participant folders with paper–pencil data are kept in a locked file cabinet located behind two locked doors, accessible only to necessary staff.

The dataset does not contain any personally identifying information. Each participant receives a unique study ID number, and the collected data is associated only with this specific number.

### Plans for collection, laboratory evaluation and storage of biological specimens for genetic or molecular analysis in this trial/future use {33}

Urine samples are collected from each participant on the screening visit (t0), intervention visit I and II (t1 and t2), and follow-up visit (t3). Blood samples are collected to screen for participants’ health during the screening visit (t0). Further, two blood samples for the BDNF analysis during intervention visit I (t1) are collected.

All results of the urine and blood analysis are done by the in-house laboratory. The urine and blood samples are disposed of as per policy guidelines by the in-house laboratory immediately after analysis.

Results of the urine and blood analysis are entered by the study team into the data management system and the source data is stored in the physical participant folder. Furthermore, the blood sample for the BDNF analysis obtained during intervention visit I (t1) are being properly prepared and stored in a freezer at −80 °C. These samples are kept under these conditions until the study is completed, at which point they will be analysed by the in-house research laboratory. Only samples of participants, who signed the separate consent form for the further assessment of biomarkers in blood plasma samples, are taken and stored after the planned BDNF analysis for further analysis.

## Statistical methods

### Statistical methods for primary and secondary outcomes {20a}

The central hypothesis that the active treatments reduce the “proportion of cocaine use days” is tested by implementing an analysis of covariance (ANCOVA) with the between-subjects factor treatment groups (i.e. rt-fMRI NFT/placebo, rt-fMRI NFT/ketamine, sham NFT/placebo, and sham NFT/ketamine) once for each treatment (i.e. NFT and ketamine) for the follow-up visit (t3). The initial ANCOVA includes variables that potentially differ between groups at baseline as potential confounding variables, namely age, gender, substance use, and severity of dependence. To retain the simplest model, all the baseline variables that do not contribute to the outcome variable ‘proportion of cocaine use days’ are excluded from the model. Should none of the baseline variables that differ between groups contribute to the outcome variable, an analysis of variance (ANOVA) is performed. Post-hoc tests are adequately corrected for multiple comparisons.

Changes in glutamate levels in the NAcc due to the ketamine infusion on intervention visit I (t1) are analysed by applying an ANOVA for repeated measurements as not many dropouts for one of the pre-ketamine and post-ketamine glutamate assessments are expected. The main source of dropouts is expected to be of technical nature, causing insufficient data quality of the ^1^H-MRS, while dropout due to lack of compliance between the two measurements is less plausible since they take place on the same day. The ANOVA for repeated measurements includes the between-group factor treatment group (i.e. rt-fMRI NFT/placebo, rt-fMRI NFT/ketamine, sham NFT/placebo, and sham NFT/ketamine) and the within-group factor time (pre-ketamine infusion vs. post-ketamine infusion). Post-hoc test is adequately corrected for multiple comparisons. To test for sustained effects of the ketamine infusion on glutamate concentrations in the NAcc between intervention visit I (t1) and intervention visit II (t2), a multilevel analysis is applied that allows to (i) analyse multiple events in one model, (ii) include subjects with incomplete data sets, (iii) account for different time intervals between the events (7 to 14 days).

The transfer effect of the reward imagery rt-fMRI NFT from the first to the second session is analysed by comparing the percent signal change in the practice run from the first reward imagery rt-fMRI NFT session (t1) and the transfer run from the third reward imagery rt-fMRI NFT session (t2) in an ANOVA for repeated measurements with a between-subject factor treatment group (i.e. rt-fMRI NFT/placebo, rt-fMRI NFT/ketamine, sham NFT/placebo, and sham NFT/ketamine) and the within-group factor time (first session at t1 vs. third session at t2). Post-hoc tests are adequately corrected for multiple comparisons. To test the impact of fatigue or of earlier reward imagery rt-fMRI NFT success on the transfer effect, an exploratory analysis with a multilevel analysis is performed that enables to include multiple events, in this case the transfer run from the first reward imagery rt-fMRI NFT session and the practice run from the third reward imagery rt-fMRI session, thereby accounting for different time intervals between those four events.

### Interim analyses {21b}

No interim analyses are planned because (a) it is not a safety measurement and (b) no statement to clinical efficacy can be made.

### Methods for additional analyses (e.g. subgroup analyses) {20b}

To test whether effects observed in the analyses of the primary outcome depend on the severity of CUD, affect, and stress, the correlations between those measures are assessed. Post-hoc tests are adequately corrected for multiple comparisons.

### Methods in analysis to handle protocol non-adherence and any statistical methods to handle missing data {20c}

The primary efficacy analysis is done using the last observation carried forward (LOCF) approach on the intention-to-treat population (ITT), i.e. all participants randomised who received ketamine treatment and reward imagery rt-fMRI NFT and had at least one assessment of efficacy 35 days post-treatment are analysed. Drop-outs before the primary efficacy assessment 35 days post-treatment are replaced. The Observed Cases (OC) approach, in which only the observed values are analysed, is used for supportive analyses.

### Plans to give access to the full protocol, participant-level data and statistical code {31c}

Access to the final trial dataset is only given to the study investigators, data monitoring committee and any other relevant regulatory bodies. De-identified databases and the full study protocol are accessible upon publication of the trial results on a database fulfilling FAIR (Findable Accessible Interoperable Reusable) principles.

## Oversight and monitoring

### Composition of the coordinating centre and trial steering committee {5d}

The Trial Steering Committee (TSC) comprises all authors listed and provides overall supervision of the trial. The TSC develops and collectively approves changes to the study protocol. Furthermore, it oversees the progress of the trial, adherence to the protocol, participant safety, and is responsible for publication and dissemination. The study team has expertise in relevant fields, including psychology, psychiatry, medical care, pharmaceutical formulation, safety assessment, conduction of clinical trials, neuroscience, and statistical analysis.

### Composition of the data monitoring committee, its role and reporting structure {21a}

Off-site data monitoring is done by an accordingly trained person independent of the sponsor-investigator. Routine data monitoring visits are scheduled according to the number of enrolled participants; in total, six monitoring visits take place, including a site initiation visit before the enrolment of the first participant, four visits during the study duration, and one close-out visit after the last enrolled participant finished the last study visit and database lock. A report is sent to the sponsor-investigator after every one of the six routine monitoring visits.

### Adverse event reporting and harms {22}

All observed or volunteered abnormal safety endpoints are recorded in the data management system. The investigator promptly reviews documented adverse events (AE) or other abnormal safety endpoints to determine if (i) the abnormal safety endpoint should be classified as an AE, (ii) if there is a reasonable possibility that the AE was caused by the investigational drug, and (iii) if the AE meets the criteria for a serious AE (SAE). The investigator is responsible for SAE reporting to the sponsor-investigator, who will conduct a documented evaluation. Additionally, all SAE must be reported to the local Ethics Committees of Zurich and Swissmedic by the sponsor-investigator.

### Frequency and plans for auditing trial conduct {23}

In total, four routine data monitoring visits are planned, where the designated person reports on participant status, current qualification of the study team, regulatory aspects, correct obtainment of informed consent, status of the eCRF, source data verification, SAE reporting and documentation, drug accountability and storage, laboratory aspects, evaluation of facilities and equipment, status of trial master file, and finally, protocol deviations.

### Plans for communicating important protocol amendments to relevant parties (e.g. trial participants, ethical committees) {25}

All substantial protocol modifications must be approved by the Ethics Committees of Zurich before implementation in the study unless the safety of the participants is at risk. Furthermore, protocol modifications are communicated to all relevant on-site members of the study team.

### Dissemination plans {31a}

The results of this clinical trial will be submitted to peer-reviewed journals for publication and will also be presented (oral or poster presentations) at international and national conferences. Both positive and negative findings will be disclosed.

## Discussion

This clinical study has the potential to constitute an urgently needed major advancement for the treatment of CUD by proposing a novel individualised and integrated pharmaco-psychotherapeutic intervention. This intervention is deeply rooted in the latest understanding of the neurobiological adaptations associated with CUD as derived from animal models [20, 21, 80] and clinical trials [19, 81–83]. We expect that the proposed pharmacological intervention will take preclinical molecular insights from bench to bedside and boost the development of novel pharmacotherapies for CUD and potentially other SUD by establishing glutamate signalling as a validated target for SUD therapy in humans. Our neuroimaging assay will allow for quantifying target engagement (i.e. the capacity of ketamine to restore glutamate signalling) together with its impact on substance craving and taking on an individual level, therefore paving the way for personalised treatment approaches by establishing accumbal glutamate concentration as a predictive biomarker. Moreover, allowing individuals with CUD to specifically restore the sensitivity of their maladaptive reward system to non-substance related reinforcers by means of rt-fMRI NFT assisted mental reward imagery constitutes a novel, very specific and highly individualised approach for psychotherapy of CUD and, if successful, also for other SUD. In addition, by combining pharmacologically induced neuroplasticity with rt-fMRI NFT-guided learning, we aim to boost the effect of the stand-alone interventions that are ideally suited to complement each other. Therefore, the findings from this study have the potential to foster novel treatments for other neuropsychiatric diseases (e.g. depression, posttraumatic stress disorder, anxiety disorders) as well that could benefit from synergistic effects achieved by combining rt-fMRI NFT-guided learning and pharmacologically boosted neuroplasticity.

### Trial status

Protocol version 2.4. was approved on December 10th, 2024. Recruitment is ongoing and started in February 2024 and will last approximately until spring 2026. At the time of this submission, three participants have already completed the study.

## Supplementary Information

Additional file 1: SPIRIT checklist

Additional file 2: Table 1: Questions between reward imagery rt-fMRI NFT runs. Table 2: Questionnaire after reward imagery rt-fMRI NFT. Table 3: Primary, secondary, other and safety outcomes

Additional file 3: Model consent form

## Data Availability

Access to the final trial dataset is only given to the study investigators, data monitoring committee, and any other relevant regulatory bodies. De-identified databases and the full study protocol are accessible upon publication of the trial results on a database fulfilling FAIR principles.

## Abbreviations

^1^H-MRS: Proton magnetic resonance spectroscopy
5D-ASC: 5-Dimension Altered State of Consciousness Questionnaire
AAL: Automated anatomical labelling atlas
AE: Adverse event
AMPA: Alpha-amino-3-hydroxy-5-methyl-4-isoxazolepropionic acid
ANCOVA: Analysis of covariance
ANOVA: Analysis of variance
AUC: Area under the curve
BDI: Beck’s Depression Inventory
BDNF: Brain-derived neurotrophic factor
BMI: Body mass index
CUD: Cocaine use disorder
DOPS: Domains of Pleasure Scale
DSM-5: Diagnostic and statistical manual of mental disorders, 5th edition
(e)CRF: (Electronic) case report form
ECG: Electrocardiogram
EMA: Ecological momentary assessment
FAIR: Findable accessible interoperable reusable
GCP: Good clinical practice
HMS: Hood’s Mysticism Scale
ITT: Intention to treat
LOCF: Last observation carried forward
LTD: Long-term depression
LTP: Long-term potentiation
MANOVA: Multivariate analysis of variance
MINI: Mini-international neuropsychiatric interview
MR: Magnetic resonance
NAcc: Nucleus accumbens
NFT: Neurofeedback training
NMDA: N-methyl-D-aspartate
NMR: Negative mood regulation expectancies scale
OC: Observed cases
OCCUS: Obsessive compulsive cocaine use scale
PFC: Prefrontal cortex
PIT: Prospective Imagery Task
rt-fMRI: Real-time functional magnetic resonance imaging
RSES: Rosenberg Self-Esteem Scale
SAE: Serious adverse event
SN: Substantia nigra
SUD: Substance use disorder
T1: T1 weighted image
THC: Trait Hedonic Capacity Scale
TSC: Trial steering committee
VTA: Ventral tegmental area
