# Supplementary material for: Co-Boost: boosting and guiding neuroplasticity by combining ketamine with neurofeedback-assisted learning—towards an individualised and integrated pharmaco-psychotherapy for cocaine addiction: study protocol for a randomised, placebo-controlled, double-blind, parallel-group, single-centre trial
Source: Trials. 2025 Sep 25;26:354. doi: 10.1186/s13063-025-08982-9 (PMC12465696; doi:10.1186/s13063-025-08982-9)
Supplement: Supplementary file 2 — Additional file 2: Table 1: Questions between reward imagery rt-fMRI NFT runs. Table 2: Questionnaire after reward imagery rt-fMRI NFT. Table 3: Primary, secondary, other and safety outcomes [file 13063_2025_8982_MOESM2_ESM.pdf]

**Table 1:** Questions between reward imagery rt-fMRI NFT runs

| Topic of question | Specific Questions |
|---|---|
| feelings and motivation | How did you feel during this round? |
| | Were you able to concentrate well on the neurofeedback training? |
| | How tired were you overall during the neurofeedback? |
| strategies | Which strategy(ies) did you use? |
| | And which strategy worked best for you? |
| contingency between feedback and strategy | What is your impression of how well you have managed to upregulate your brain activity |
| looking at the cross during | Did you think about other things while the cross was being shown? |

**Table 2** : Questionnaire after reward imagery rt-fMRI NFT

| Topic of question | Specific Questions |
|---|---|
| liking and ability of NFT | How much did you enjoy the neurofeedback training? |
| | How easily did suitable strategies come to your mind? |
| | How difficult was it to upregulate your brain activity using your strategies? |
| strategies | Which strategies did you use? |
| | And how well did the strategies work? |
| substance-related or music-related strategies | Did you have to think about cocaine or other substances? |
| | Did you nonetheless use music as strategy? |
| improvement | How much has your ability to upregulate your brain activity improved because of the neurofeedback training? |

**Table 3:** Primary, secondary, other and safety outcomes

| Domaine | Measure |
|---|---|
| **Primary outcomes** | |
| Cocaine use (self-report) | Timeline Follow Back for cocaine use |
| Glutamate changes pre to post infusion on t1 | $^{1}$H-MRS scan |
| rt-fMRI NFT performance differences from t1 to t2 | fMRI scan |
| **Secondary outcomes** | |
| Cocaine use (objective measure) | Urine analysis |
| Cocaine craving during the past weeks | Obsessive Compulsive Cocaine Use Scale |
| Severity of cocaine use disorder | Obsessive Compulsive Cocaine Use Scale |
| Motivation for change regarding cocaine use behavior | Visual analogue scale for change |
| Reward sensitivity: ability to experience pleasure | Trait Hedonic Capacity Scale |
| Reward sensitivity: assessment of levels of pleasure across different domains | Domains of Pleasure Scale |
| Emotion regulation skill | Negative Mood Regulation Expectancies scale |
| Depressive symptoms | Beck Depression Inventory |
| Stress coping | Subscale of the Stress & Coping inventory |
| Self-esteem | Rosenberg self-esteem scale |
| Self-efficacy | Questionnaire for Self-efficacy, optimism, and pessimism |
| Subjective effects of infusion: changes in cognition and perception | 5-Dimension Altered State of Consciousness Questionnaire |
| Subjective effects of infusion: mystical experiences | Hood's Mysticism Scale |
| Vividness of mental imagery | Prospective Imagery Task |
| Individual imaging strategies | Neurofeedback Post Questionnaire |
| Individual imaging strategies | Neurofeedback in between Questionnaire |
| Current cocaine craving during study visits | Visual analogue scale for craving |
| Current affective state during study visits | Visual analogue scale for affective state |

| | |
|---|---|
| Glutamate changes during craving paradigm | $^{1}H$-MRS scan |
| BDNF | Enzyme-linked assay |
| Cocaine use in everyday life | Ecological momentary assessment |
| Cocaine craving in everyday life | Ecological momentary assessment |
| Affective state in everyday life | Ecological momentary assessment |
| Reward sensitivity in everyday life | Ecological momentary assessment |
| **Other outcomes of interest** | |
| Cardiac electrical activity on t0 and during ketamine/placebo infusion on t1 | Electrocardiogram |
| Psychiatry disorders | Mini-International Neuropsychiatric Interview, DSM-5 |
| Use of psychoactive substances | Interview for Psychotropic Drug Consumption |
| Subjective experience of intervention | Qualitative interview |
| **Safety outcomes** | |
| Vital signs | Blood pressure monitor |
| Concomitant medication | Questionnaire for concomitant medication |
| Adverse events | Question about adverse events |
| Current Suicidality | Suicidality screening questions from Mini-International Neuropsychiatric Interview |
| Pregnancy | Urine pregnancy test |
| Drug use | Urine drug test |
| Routine laboratory parameters | Blood analysis |
