# Supplementary material for: Co-Boost: boosting and guiding neuroplasticity by combining ketamine with neurofeedback-assisted learning—towards an individualised and integrated pharmaco-psychotherapy for cocaine addiction: study protocol for a randomised, placebo-controlled, double-blind, parallel-group, single-centre trial
Source: Trials. 2025 Sep 25;26:354. doi: 10.1186/s13063-025-08982-9 (PMC12465696; doi:10.1186/s13063-025-08982-9)
Supplement: Supplementary file 3 — Additional file 3: Model consent form [file 13063_2025_8982_MOESM3_ESM.pdf]

Request to Participate in Medical Research:

Ketamine and Neurofeedback Training: Effects on Neuroplasticity in the Treatment of Cocaine Addiction

Dear Sir or Madam,

We are asking if you would be willing to participate in our research project.

Your participation is voluntary. All data collected in this project is subject to strict data protection regulations. The research project is conducted by PD Dr. med. Marcus Herdener. If you are interested, we will gladly inform you about the results of the research.

In a meeting, we will explain the key points and answer your questions. Here's an overview of the most important information:

Why are we conducting this research project?

- Previous studies have shown that cocaine use disorder is associated with changes in brain metabolism.
- This clinical study aims to examine whether a combination of a single dose of ketamine and neurofeedback training can restore these neurobiological changes and what effects this new treatment approach might have on cocaine use disorder symptoms.

What does participation involve? – What happens to me during participation?

- If you decide to participate, you will be randomly assigned to one of four groups. You will receive either ketamine or a placebo in combination with neurofeedback training, which may be either real or sham feedback regarding your brain activity.
- The study dates are distributed over a time period of approximately 6 months.
- The study participation consists of six appointments, out of which four are held in person at the Psychiatric University Clinic Zurich, one is held online or via telephone and one is carried out as an online survey. The appointment durations are 3.5h, 7h, 5h, 2h, 1h and 1h respectively.

What are the benefits and risks of the study?

Benefits

- You will not receive any direct proven benefit from participating in this research project.
- However, there are scientific indications that treatment with ketamine and/or neurofeedback training could have positive effects regarding symptoms of cocaine use disorder (e.g. reduction in use). However, this has not yet been clearly proven.
- By participating, you help future patients by contributing to the development of new therapies.

Risks and Burdens

- Ketamine is a safe and well-researched drug from the group of anesthetics. At a dosage of ketamine as used in the study, temporary nausea, vomiting, dizziness, increased sweating and elevated pulse and blood pressure may occur. Possible psychological side effects include temporary psychotic symptoms (e.g., feeling detached from reality or being in a dream, hallucinations), altered body perception, reduced pain sensitivity, and increased anxiety.
- Neurofeedback training, in which you learn to consciously control the activity of specific brain regions, is conducted with the help of a magnetic resonance imaging (MRI) scanner. The MRI examination is a modern, extensively tested and established procedure that is carried out with specially designed equipment and does not pose any health risks to participants. According to current knowledge, the MRI examination poses no risk to human health.

By signing at the end of the document, you confirm that you are participating voluntarily and that you have understood the content of the entire document.

Detailed Information

1. Objective and Selection

We refer to our research project in this information sheet as a *study*. If you participate in this study, you are a *study participant.*

Before a drug is used for a disorder, it must be scientifically examined on study participants. We therefore want to investigate whether and how well the drug ketamine in combination with neurofeedback training works in the treatment of cocaine use disorder.

Ketamine is an anesthetic developed in 1964, primarily used in emergency medicine. It was later discovered that ketamine also has antidepressant effects. Based on this, over the past 20 years, a large body of scientific studies has emerged investigating the effects of ketamine on other psychiatric disorders. In Switzerland, ketamine is occasionally used outside the approved indication (off-label use), i.e., not for anesthesia, for the treatment of alcohol and cocaine use disorder.

In neurofeedback training, study participants learn how to influence the activity of certain brain regions through mental imagination. In this study, brain activity is recorded using functional magnetic resonance imaging (fMRI) and reported back to the study participants in real time. In this way, participants immediately receive feedback indicating how active a specific brain region is.
Often, in people with cocaine use disorder, sensitivity to positive experiences not associated with drug use is reduced. The aim is for mental imagination of positive events to deliberately promote the activation of brain regions associated with reward. In addition, the training can lead to an enhanced experience of positive events in everyday life. This is an important factor in the treatment of addictive disorders.

In this study, we would like to investigate both the sole effects of ketamine therapy and neurofeedback therapy on the symptoms of cocaine use disorder, as well as the combination of the two treatments approaches. Through MRI measurements and blood analyses, we want to learn more about the biological background of cocaine use disorder, thereby enabling the development of improved treatment approaches.

People eligible for participation include those with cocaine use disorder between the ages of 18 and 55. You should be willing to refrain from using illegal substances for three days before the second appointment and refrain from drinking alcohol for two days before this appointment. You must be in good physical condition, and if you are taking psychotropic medications, the medication must not change during the course of the study. You should speak and understand (Swiss) German well and be right-handed. Women of childbearing age must use sufficient pregnancy prevention during the study (e.g., condoms, diaphragm, hormonal pill, depot injection, intrauterine device (IUD)).

You may not participate if you have shown allergic or hypersensitivity reactions or other side effects from previous ketamine use. Individuals with severe mental or physical illness will be excluded from the study. Pregnancy or breastfeeding is also a reason for study exclusion. Certain safety regulations apply for MRI scans, which is why people with contraindications for MRI (e.g., pacemakers, metal fragments, large tattoos) cannot participate.
If any of these points apply to you, please inform the study management.

2. General Information

- The study takes place at the Psychiatric University Hospital Zurich.
- The study includes participation in six appointments. These six appointments are spread over approximately 6 months and last 3.5h, 7h, 5h, 2h, 1h, and 1h respectively.
- Between the first five appointments, we will ask you questions about your current cocaine use and craving via an app on your smartphone and offer exercises in mental imagery.

- The qualitative interviews at the 2nd, 3rd, and 4th appointments will be audio recorded for later analysis.
- This is a placebo-controlled study. That means both the ketamine and the neurofeedback training will be compared with a control substance or control training. For ketamine, the control substance is a saline solution (placebo), and for the neurofeedback training, if you are assigned to the control group, you will receive fictitious feedback (sham) instead of real feedback.
- The study consists of four groups in which participants receive different combinations of the two interventions: 1) ketamine and neurofeedback training with real feedback 2) placebo und neurofeedback training with real feedback 3) placebo und sham neurofeedback, as well as 4) ketamine and sham neurofeedback.
- You will be randomly assigned to one of these four groups, with an equal probability of being placed in any group.
- Group 3 (placebo und sham neurofeedback) is a pure control group, meaning no therapeutic effects are expected here. However, previous studies suggest that even the placebo effect, the support from the study team, and the app-based questionnaires and exercises during the study may have a positive impact.
- For the administration of ketamine as well as for the neurofeedback training, neither the study team nor the participants will know whether ketamine or the placebo is being administered, or whether the participants are receiving real or sham feedback (double-blind).
- Ketamine is administered via intravenous infusion, i.e., through a vein access. The dosage is adjusted to your body weight and is 0.71 mg/kg. The method of administration and dosage have already been used in previous studies and show good tolerability.
- A total of 120 participants with cocaine addiction will be examined.
- The study runs from October 2023 to December 2025.
- We are conducting this study as required by Swiss law. In addition, we follow all internationally recognized guidelines. The responsible cantonal ethics committee and Swissmedic have reviewed and approved the study.
- A description of our study can be found on the website of the Federal Office of Public Health: www.kofam.ch (registration number SNCTP000005678).

3. Procedure

- The first four of the six study appointments will take place at the Psychiatric University Hospital Zurich. During the first appointment, it will be checked whether you meet all the criteria for study participation. On the second appointment, you will receive either a placebo or ketamine, and we will conduct the neurofeedback training with real or sham feedback. The neurofeedback training will be repeated at the third appointment. At the fourth appointment, you will answer some questionnaires on site. The fifth appointment will take place as an online meeting or by telephone. Here, we would like to ask you about your current consumption behavior. The sixth and final appointment will take place as an online survey. At each of the six appointments, we would like to ask you questions about your well-being and consumption behavior. Between the first five appointments — that is, four times in total — we will send you questions via an app on your smartphone regarding your current well-being and consumption behavior, and we will provide exercises. Figure 1 shows the entire study procedure in a graphic:

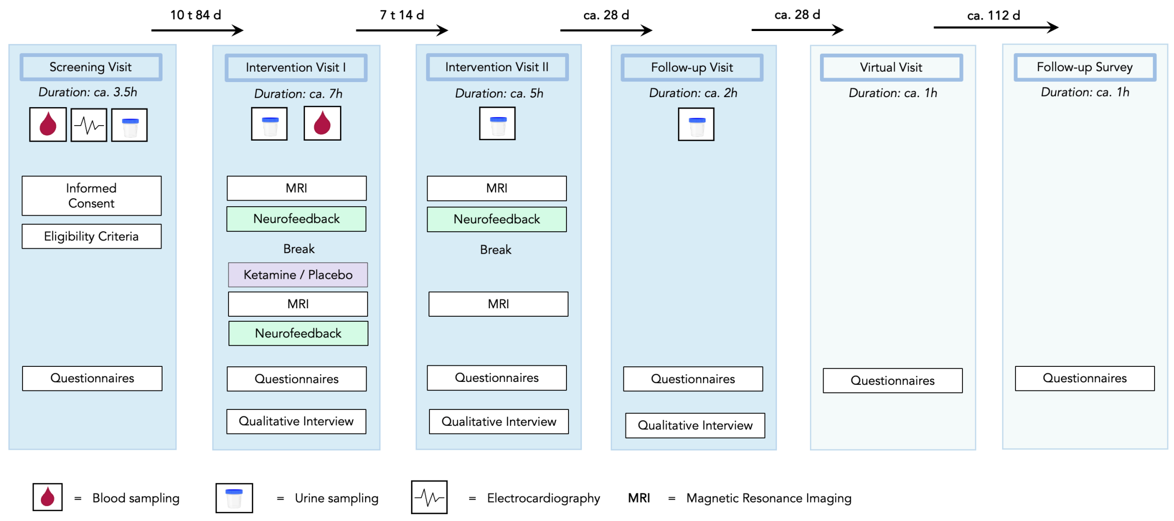

Figure 1: Procedure of the six study appointments. The first four appointments take place on-site at the Psychiatric University Hospital Zurich. The fifth appointment takes place as an online meeting or by telephone, and the sixth appointment is an online survey that you can complete from home.

- 1st Appointment (Duration approx. 3.5h): At the first appointment, we will check whether you meet all the criteria for study participation. The medical clarification includes the electrical recording of heart activity (electrocardiogram = ECG) as well as a blood sample (25 ml). Through blood analysis, we want to examine whether there are changes in the concentration of nerve cell growth proteins during the course of the study. Current cocaine/drug use will be measured via a urine analysis. Additionally, we will conduct an interview regarding your medical and psychiatric history and ask you to answer further questions about your mental and physical health as well as your consumption behavior.
- 2nd Appointment (Duration approx. 7h): The intervention takes place earliest ten days after the first appointment. First, you will provide a urine and blood sample (10 ml), and for women of childbearing age, an additional urine sample for a pregnancy test will be collected. Next, we will perform a so-called baseline measurement via MRI to record your baseline condition. Additionally, you will then receive your first neurofeedback training with real or sham feedback. Afterward, you will receive a placebo or ketamine, followed by further MRI scans and another neurofeedback training session. After the infusion, we will again take another blood sample (10 ml) to examine the possible influence of ketamine on plasticity markers. Finally, you will answer some questionnaires on your well-being and consumption behavior, and we will conduct a qualitative interview about the session. This interview will be audio recorded.
- 3rd Appointment (Duration approx. 5h): The third appointment takes place approximately 7 days after the second appointment. At the beginning, current cocaine/drug use will be measured via a urine analysis, and for women of childbearing age, an additional urine sample for a pregnancy test will be collected. Then, another neurofeedback training will take place, including the associated MRI scan. Additionally, we will again ask you questions about your well-being and consumption behavior and conduct a qualitative interview, which will be audio recorded.
- 4th Appointment (Duration approx. 2h): The fourth appointment takes place approximately 28 days after the third appointment. Here, you will answer questions about your well-being and consumption behavior and provide a urine sample so we can measure current cocaine/drug use. We will also conduct a qualitative interview, which will be audio recorded.
- 5th Appointment (Duration approx. 1h): The fifth appointment takes place approximately 28 days after the fourth appointment, online or by telephone. We would like to ask you about your current well-being and consumption behavior.

- 6th Appointment (Duration approx. 1h): The sixth and final appointment takes place approximately 6 months after the second appointment. It is conducted in the form of an online survey in which we will ask you questions about your well-being and consumption behavior.
- Interim surveys using the SEMA^3 app on smartphone: Between the first five appointments — that is, four times in total — we would like to ask you about your current cocaine use and craving on seven consecutive days. You will receive these questions via push notifications through the SEMA^3 app. Additionally, after the neurofeedback training, you will also have the opportunity within the app to do mental imagery exercises related to positive events. We will explain the use and installation of this app to you in advance.

It may happen that you wish or need to terminate your participation early**.** This can occur if you withdraw your consent to participate in the study during the course of the study (which is possible at any time), if continuing participation poses a health risk to you, or if you do not comply with the study regulations. In the event of an early study termination, we will offer you a final examination for your safety.

4. Benefits

You may not personally benefit from participating in the study. However, previous studies suggest that treatment with ketamine and/or neurofeedback training with real feedback could have a positive effect on cocaine use disorder. These effects, however, have not yet been systematically investigated and have yet to be scientifically proven. The results of this study may be important in the future for other individuals with cocaine use disorder, as they are intended to help develop more effective treatment approaches for this disorder.

5. Voluntariness and Obligations

You are participating in this study voluntarily. If you do not wish to participate in this study or later wish to withdraw, you do not have to provide a reason. Your medical care is guaranteed regardless of your decision.

If you participate in this study, you are asked:
- to comply with the requirements and demands of the study according to the study protocol. This includes, among other things, the willingness to refrain from consuming illegal substances for three days and from consuming alcohol for two days prior to the second appointment.
- to inform the study management or the study physician about the course of the condition and report new symptoms, new complaints, and changes in your condition.
- to inform the study management or the study physician about any concurrent treatment and therapy by other doctors and about the intake of medications.
- not to drive a vehicle or operate machinery on the day of the second appointment (after ketamine administration).

If you do not fulfill these obligations, you may lose your entitlement to liability claims.

6. Risks and Burdens

Risks and burdens from ketamine:
We are using Ketalar®, which is usually used for anesthesia and analgesia and is administered in those areas at a higher dosage. In our study, we will use lower, so-called sub-anesthetic dosages (without inducing anesthesia/narcosis). The following are undesired effects that may occur temporarily with a sub-anesthetic dosage:

- Nausea
- Vomiting

- Dizziness
- Increased sweating
- Increased heart rate
- Increased blood pressure
- Feeling detached from reality or like being in a dream
- Hallucinations
- Altered perception of the body
- Reduced pain sensation
- Increased anxiety

Some individuals report a "hangover feeling" the next day and vivid dreams a few days after the ketamine administration.

Risks and burdens from countermedications:
In very rare cases, to treat the side effects of ketamine, a medication approved for the corresponding symptoms and frequently used may be administered. The risks and burdens associated with the countermedication are briefly listed below:

- **Adalat retard®** (in case of high blood pressure): may cause headaches, constipation, vasodilation, or soft tissue swelling.
- **Temesta®** (in case of severe anxiety or agitation): may cause drowsiness, dizziness, weakness, and in rare cases, breathing difficulties, respiratory arrest, or anaphylaxis.
- **Zyprexa®** (in case of psychotic symptoms such as delusions, disorganized behavior, or aggression): may cause drowsiness, dizziness, blood pressure drop, motor disturbances, dry mouth, tremors, nausea and vomiting, and in rare cases, seizures, malignant neuroleptic syndrome, and tardive dyskinesia.
- **Zofran®** (in case of nausea): may cause headaches and hot flashes. In rare cases, it may cause movement disorders, cardiac arrhythmias, angina pectoris, slowed heartbeat, and constipation.

Risks and burdens from the magnetic resonance imaging (MRI) scan:
MRI is a modern, extensively tested, and established procedure that is carried out with specially designed equipment and poses no health risk to participants. According to current knowledge, MRI scanning poses no health risk to humans. Known hazards only arise from metallic objects or electronic implants in the body. Due to the technically required magnetic field, one must not approach the device with metallic objects. You must remove all metallic items before the examination. This includes all metal body jewelry (piercings, earrings, etc.). Please also inform the study doctor or management about any tattoos. Since tattoos may contain metallic particles, they can heat up during MRI and potentially cause burns. Individuals with large tattoos are therefore excluded from the study. Also excluded are individuals with metal parts in the body (vascular clips, artificial heart valves, pacemakers, nerve stimulators, Swan-Ganz catheters, insulin pumps, cochlear implants, metal prostheses, also metal fragments from gunshot wounds or metal splinters in the eye, work in the metal industry).
An MRI safety screening is therefore carried out before every examination. Because the MRI scanner tube offers little space due to technical reasons and participants must lie inside the tube during the scan, a feeling of tightness up to claustrophobia may occur. Individuals prone to claustrophobia are not allowed to participate in the study. Scanner devices with the magnetic field strength used in this study (3.0 Tesla) have been regularly used in hospitals for routine diagnostics for many years, and no health effects have been found. Still, avoid rapid movements in the magnetic field; they may temporarily cause dizziness or a metallic taste in the mouth. Occasionally, brief flashes of light, so-called phosphenes, are perceived. The radio waves used in MRI are subject to similar limit values as those for mobile phones, which are strictly adhered to during MRI examinations. This ensures that potential effects on the body are avoided. To protect against the knocking noises during the scan, you will receive hearing protection compliant with occupational safety regulations. You can still contact the staff at any time via intercom.

Risks and burdens from blood draws:
Blood draws are carried out by medically trained personnel. You will have one 25 ml and two 10 ml blood samples (venous) taken. Risks associated with blood draws include the development of a bruise, redness and/or swelling at the site of the draw, as well as local infection. When performed properly, however, these risks are very low.

For Women Who Can Become Pregnant
There are no controlled studies in pregnant women that could provide information about possible consequences for the unborn child. Therefore, for women of childbearing age, a pregnancy test will be carried out before the start of the study as well as at the following appointments. To ensure that participants do not become pregnant during the study, a reliable method of contraception must be used (e.g., condoms, diaphragm, hormonal pill, depot injection, intrauterine device (IUD)).

Should you become pregnant during the study, you must immediately inform your study management or study doctor and may no longer participate in the study. In this case, you will be asked to provide information about the course and outcome of the pregnancy. The study management or study doctor will discuss the next steps with you.

If you are breastfeeding, you are excluded from participation.

7. Alternatives
Participation in the study involves both opportunities and risks. If you do not participate, your treating physician can arrange for psychotherapy.

8. Results
There are
1. individual results from the study that directly concern you,
2. individual results from the study that arise incidentally (so-called incidental findings),
3. objective final results of the overall study.

1: Your study physician will inform you during the study of all new results and findings that are personally important to you. You will be informed verbally and in writing and can then decide again whether you wish to continue participating in the study.

2: Incidental findings are so-called “accompanying results,” meaning findings that were not explicitly sought but were discovered by chance. These may result from imaging procedures (MRI scans) or blood analyses, for example. If incidental findings are relevant to your health, you will be informed. This means that you will be notified if a previously unknown illness is detected by chance, or if a condition not yet manifested can potentially be prevented through early intervention. Appropriate medical counseling will be ensured. If you do not wish to be informed about such findings (known as the “right not to know”), please inform your study physician accordingly.

3: Your study physician can provide you with a summary of the overall results at the end of the study.

9. Confidentiality of Data and Samples
9.1. Data processing and Encryption
For this study, personal and health-related data about you will be collected and processed, partly in automated form. During data collection, your data will be encrypted. Encryption means that all identifying information (name, date of birth, etc.) will be removed and replaced with a code. People who do not have access to this key list cannot trace the data back to you. The key list remains stored at the institution at all times.
Only a very small number of professionals will see your unencrypted data — and only to carry out tasks within the framework of the study. These individuals are bound by professional confidentiality. As a participant, you have the right to access your data.

The audio recordings of the qualitative interviews must be stored for legal reasons for 10 years. These recordings will be stored on the server of the Psychiatric University Hospital with physical access control (key), password-protected, and will only be evaluated by the study team itself.

9.2. Data Protection and Sample Protection

All data protection regulations will be strictly followed. It is possible that your data may be transmitted in encrypted form, for example for publication, and made available to other researchers. If health-related data/samples are stored locally, they are part of a database/biobank for research purposes.

9.3. Data Protection in Case of Reuse

Your data and samples could later be important for answering other research questions and may be sent and used at a later time in another database/biobank in Switzerland or abroad for as-yet undefined investigations. This other database/biobank must comply with the same standards as the database/biobank for this study.
To allow for such reuse, we ask you to sign an additional consent form at the very end of this document. This second consent is independent of your participation in this study.

9.4. Right of Access in Audits

This study may be audited by the responsible ethics committee, the medicinal product authority Swissmedic, or the sponsor that initiated the study. In such cases, your data must be disclosed by the study physician. All parties are required to maintain absolute confidentiality.

10. Withdrawal

You can withdraw from the study at any time. However, the data and samples collected up to that point will still be analyzed in encrypted form as part of the study. After analysis, your data and samples will be anonymized, and the samples will be destroyed. The key linking the data to your identity will also be destroyed, so that no one can subsequently determine that the data and samples originally came from you. This is primarily to protect your data privacy.

11. Compensation

If you participate in this study, you will receive compensation. This amounts to a maximum of CHF 370 for full participation (six study appointments including surveys via the app on your smartphone). In the case of early withdrawal from the study, you will receive proportional compensation. Expenses such as travel costs incurred due to participation will be reimbursed by arrangement. No costs will be incurred by you or your health insurance for participation.

12. Liability

The institution (the Psychiatric University Hospital Zurich), which initiated and is responsible for conducting the study, is liable for any damage that may occur in connection with the test substance and research procedures (e.g., examinations). The conditions and procedures for this are legally regulated.
The Psychiatric University Hospital Zurich has therefore taken out insurance with Zurich Insurance Company Ltd (Mythenquai 2, 8002 Zurich) to cover liability in the event of any damage. If you suffer harm from participating in this study, please contact the study physician or the above-mentioned insurance company.

13. Funding

The study is fully funded by the Swiss National Science Foundation (SNSF).

14. Contact Person(s)

You may ask questions about participation in the study at any time. In case of uncertainties or emergencies that occur during or after the study, please contact:

Study Physician:
Dr. med. Laurent Becciolini
Psychiatric University Hospital Zurich, University of Zurich
Department of Adult Psychiatry and Psychotherapy
Centre for Addictive Disorders
Selnaustrasse 9
8001 Zürich, Switzerland
E-mail: laurent.becciolini@pukzh.ch
Phone: +41 58 384 58 81

Study Management:
Etna Engeli, PhD
Psychiatric University Hospital Zurich, University of Zurich
Department of Adult Psychiatry and Psychotherapy
Addictive Disorders Research Group
Lenggstrasse 31
8032 Zürich
Switzerland
Email: etna.engeli@bli.uzh.ch
Phone: +41 58 384 27 71

M. Sc. Anna Trippel
Psychiatric University Hospital Zurich, University of Zurich
Department of Adult Psychiatry and Psychotherapy
Addictive Disorders Research Group
Lenggstrasse 31
8032 Zürich
Switzerland
Email: anna.trippel@bli.uzh.ch
Phone: +41 58 384 27 71

M. Sc. Ladina Gubser
Psychiatric University Hospital Zurich, University of Zurich
Department of Adult Psychiatry and Psychotherapy
Addictive Disorders Research Group
Lenggstrasse 31
8032 Zürich
Switzerland
Email: ladina.gubser@bli.uzh.ch
Phone: +41 58 384 27 71

Study Supervisor:
PD Dr. med. Marcus Herdener
Department of Adult Psychiatry and Psychotherapy
Centre for Addiction Disorders
Selnaustrasse 9
8001 Zürich, Switzerland
Email: marcus.herdener@bli.uzh.ch
Phone: +41 58 384 58 10

Emergency number, 24h availability: 0800 33 66 55

Declaration of Consent

Written Declaration of Consent to Participate in a Clinical Study
Please read this form carefully. Please ask questions if there is anything you do not understand or would like to know. Your written consent is required for participation.

**BASEC Number (after submission):**	2022-01859
**Title of Study**<br>**(scientific and lay language)**:	Ketamine and Neurofeedback Training: Effects on Neuroplasticity in the Treatment of Cocaine Addiction
**Responsible institution**<br>**(Sponsor with address):**	PD Dr. med. Marcus Herdener<br>Department of Adult Psychiatry and Psychotherapy<br>Centre for Addiction Disorders<br>Selnaustrasse 9<br>8001 Zürich, Switzerland
**Location of the study**:	Psychiatric University Hospital Zurich<br>Lenggstrasse 31<br>8032 Zurich
**Responsible study management at the study site:**<br>Name and first name in block letters:	
**Participant:**<br>Name and first name in block letters:<br>Date of birth:	

- I have been informed verbally and in writing by the undersigned study management about the purpose and the procedure of the study using the new treatment method of ketamine and neurofeedback, including potential advantages, disadvantages, and risks.
- I voluntarily participate in this study and accept the content of the written information provided to me. I had sufficient time to make my decision.
- My questions related to participation in this study have been answered. I will keep the written information and receive a copy of my written declaration of consent.
- I have been informed about possible alternatives to the study, e.g., other treatment methods.
- I agree that authorized professionals from the sponsor, the responsible ethics committee, and the regulatory authority Swissmedic may, for the purpose of audits and inspections, access my unencrypted data — under strict confidentiality.
- I will be informed of results or incidental findings that directly affect my health. If I do not wish to be informed, I will notify the study management.
- I understand that my health-related and personal data (and samples) can only be shared in encrypted form for research purposes in this study. The sponsor ensures that data protection according to Swiss standards will be upheld.
- I can withdraw from the study at any time and without giving reasons. My continued medical treatment is guaranteed, regardless of my decision. Data and samples collected before withdrawal will still be analyzed as part of the study.
- I have been informed that the Psychiatric University Hospital Zurich has taken out insurance that covers any harm resulting from the research project.

- I am aware that the obligations stated in the information sheet must be observed. In the interest of my health, the study management or study physician may exclude me from the study at any time.

Place, Date	Signature of Participant

Confirmation of the Study Management:
I hereby confirm that I have explained the nature, significance, and scope of the study to this participant. I assure that I will fulfill all obligations associated with this study in accordance with Swiss law. During the course of the study, should I learn of any aspects that could influence the participant's willingness to take part in the study, I will immediately inform them.

Place, Date	Surname and first name of the study management in block letters<br><br>Signature of the study management

Declaration of Consent for the Reuse of Data and Biological Material in Encrypted Form

**BASEC-Nummer (after submission):**	2022-01859
**Title of Study (scientific and lay language)**:	Ketamine and Neurofeedback Training: Effects on Neuroplasticity in the Treatment of Cocaine Addiction
**Participant:**<br>Surname and first name in block letters:<br>Date of birth:	

I allow my encrypted data and samples from this study to be reused for medical research purposes. The samples will be stored in a biobank and used for future, as yet undefined, research projects for an indefinite period of time.

I understand that the samples are encrypted and the key is stored securely. The data and samples may be sent to other data and biobanks in Switzerland or abroad for analysis, provided that these comply with the same standards as in Switzerland. All legal regulations regarding data protection will be followed.

I am deciding voluntarily and may withdraw this decision at any time. If I withdraw, my data will be anonymized, and my samples and data will be destroyed. I will simply inform the study management or study physician of my decision and do not have to provide a reason.

Normally, all data and samples are analyzed as a whole and the results are summarized and published. If a finding relevant to my health emerges from the data and samples, I may be contacted. If I do not wish to be informed of such findings, I will inform the study management or study physician.

I allow my data and samples to be anonymized and understand that, in this case, I cannot be informed of incidental findings and cannot withdraw from the research project anymore.

If results from the data and samples are commercialized, I will not be entitled to any share in the commercial use.

Place, Date	Signature of Participant

Confirmation of the Study Management: I hereby confirm that I have explained the nature, significance, and scope of the reuse of samples and/or genetic data to this participant.

Place, Date	Surname and first name of the study management in block letters<br><br>Signature of the study management
